# First nimravid skull from Asia

**DOI:** 10.1038/srep25812

**Published:** 2016-05-10

**Authors:** Alexander Averianov, Ekaterina Obraztsova, Igor Danilov, Pavel Skutschas, Jianhua Jin

**Affiliations:** 1 State Key Laboratory of Biocontrol and Guangdong Provincial Key Laboratory of Plant Resources, School of Life Sciences, Sun Yat-sen University, Guangzhou 510275, China; 2 Zoological Institute of the Russian Academy of Sciences, Universitetskaya Nab. 1, St. Petersburg 199034, Russia; 3 Department of Vertebrate Zoology, Biological Faculty, Saint Petersburg State University, Universitetskaya Nab. 7/9, St. Petersburg 199034, Russia

## Abstract

*Maofelis cantonensis* gen. and sp. nov. is described based on a complete cranium from the middle-upper Eocene Youganwo Formation of Maoming Basin, Guangdong Province, China. The new taxon has characters diagnostic for Nimravidae such as a short cat-like skull, short palate, ventral surface of petrosal dorsal to that of basioccipital, serrations on the distal carina of canine, reduced anterior premolars, and absence of posterior molars (M2-3). It is plesiomorphic nimravid taxon similar to Nimravidae indet. from Quercy (France) in having the glenoid pedicle and mastoid process without ventral projections, a planar basicranium in which the lateral rim is not ventrally buttressed, and P1 present. The upper canine is less flattened than in other Nimravidae. *Maofelis cantonensis* gen. and sp. nov. exemplifies the earliest stage of development of sabertooth specialization characteristic of Nimravidae. This taxon, together with other middle-late Eocene nimravid records in South Asia, suggests origin and initial diversification of Nimravidae in Asia. We propose that this group dispersed to North America in the late Eocene and to Europe in the early Oligocene. The subsequent Oligocene diversification of Nimravidae took place in North America and Europe, while in Asia this group declined in the Oligocene, likely because of the earlier development of open habitats on that continent.

Nimravids are cat-like hypercarnivores that developed saber-tooth morphology early in the Cenozoic and were top predators in the late Eocene – late Oligocene mammal communities of the Northern Hemisphere^1^,^2^,^3^. Cope^4^ considered the Paleogene nimravids as direct ancestors of the modern cats (Felidae) and this view was generally adopted in the 19th and first half of 20th centuries^5^,^6^. Piveteau^7^ thought that Nimravidae and Felidae are sister taxa. The current consensus is that Nimravidae are stem aeluroids not closely related to felids^1^,^8^,^9^,^10^. Nimravidae were traditionally divided into the late Eocene – late Oligocene Nimravinae and the middle-late Miocene Barbourofelinae^1^,^11^, but the recent data suggest that these two groups are not closely related^12^,^13^,^14^.

The fossil record of Nimravidae is excellent in North America^4^,^6^,^10^,^11^,^15^,^16^,^17^,^18^,^19^,^20^,^21^,^22^,^23^,^24^,^25^,^26^,^27^ and reasonably complete in Europe^2^,^7^,^12^,^28^,^29^,^30^,^31^,^32^,^33^. In contrast, in Asia nimravids have been known so far from few dentary and tooth fragments^34^,^35^,^36^,^37^,^38^,^39^,^40^,^41^,^42^ (Fig. 1). Here we report the first nimravid skull from Asia. This specimen was found by a local amateur collector in the upper Eocene Youganwo (=Youkanwo) Formation exposed in an oil shale quarry near Maoming, Guangdong Province, China. In 2013 the specimen was obtained by the School of Life Sciences of Sun Yat-sen University (Guangzhou, China) and subsequently prepared by PS and EO. Here we provide a preliminary description of this specimen, establish the first nimravid taxon endemic to Asia, and discuss its significance for understanding the evolutionary history of Nimravidae.

## Results

Systematic paleontology.

Mammalia Linnaeus, 1758

Carnivora Bowdich, 1821

Nimravidae Cope, 1880

*Maofelis cantonensis* gen. et sp. nov.

Figures 2, 3, 4; Supplementary Figures 1, 2, 4–6.

Etymology: The generic name is from Maoming Basin in Guangdong Province where the skull was found, and the felid genus *Felis*. The species name is from Canton, an older name of Guangzhou City.

Holotype: Collection from the Maoming Basin in the School of Life Sciences, Sun Yat-sen University, Guangzhou, China (SYSU-M) 2, almost complete cranium (skull without mandible), with most of the dentition preserved.

Type locality and horizon: The oil shale quarry (21°42′ N, 110°53′ E) located near Maoming City, Maoming Basin, Guangdong Province, China; Youganwo Formation, middle-upper Eocene.

Diagnosis: *Maofelis* is referred to Nimravidae based on the following combination of characters that is diagnostic for this group^1^,^2^: cat-like skull with shortened rostrum and mesocranium; palate short, does not extend posterior to the toothrow; walls of the basipharyngeal canal converged posteriorly; ventral surface of petrosal significantly dorsal to that of basioccipital; hypoglossal foramen separated from the posterior lacerate foramen; paraoccipital process moderately large, posteriorly projecting; incisors with reduced lingual cingula; serrations on the distal carina of canine; anterior premolars reduced; P4 without a parastyle; posterior molars (M2–3) absent.

Differential Diagnosis: *Maofelis* is similar to Nimravidae indet. from Quercy (Muséum d’Histoire naturelle de Montauban, France (MA-PHQ) 348) and differs from the remaining nimravids in having no ventral projection of the glenoid pedicle or mastoid process, a planar basicranium with the lateral rim not or weakly buttressed ventrally, and P1 present (P1 is variably present in *Nimravus* and *Eofelis*). *Maofelis* differs from all known nimravids except Nimravidae indet. from Quercy (MA-PHQ 348) and *Dinaelurus* in having upper canines that are only slightly flattened (length/width ratio ~1.3); from *Nanosmilus*, *Hoplophoneus*, and *Eusmilus* by having sutural contact between the jugal and the lacrimal; from *Nimravus*, *Dinictis*, *Hoplophoneus*, and *Eusmilus* by the presence of a strongly developed sagittal crest; from *Hoplophoneus* and *Eusmilus* by the presence of a sagittal crest that is rectilinear (not concave) in lateral view; from *Nimravus* and *Dinaelurus* by the lack of a fossa on the ventromedial face of the zygomatic arch, below the postorbital process; from *Dinaelurus*, *Nanosmilus*, *Hoplophoneus*, and *Eusmilus* by the presence of a double-rooted P2; from *Dinaelurus*, *Nimravus*, *Pogonodon*, *Dinictis*, *Nanosmilus*, *Hoplophoneus*, and *Eusmilus* by the presence of a three-rooted P3; from *Hoplophoneus* and *Eusmilus* by the P4 protocone projecting strongly mesiolingually, with its mesial end at or anterior to the mesial end of the paracone; from *Dinaelurus*, *Nimravus*, *Nanosmilus*, *Hoplophoneus*, and *Eusmilus* by the presence of a transversely elongated M1, in which the protocone is prominent and widely separated from the paracone; from *Dinaelurus*, *Nimravus*, and *Eusmilus* by the M1 crown not being vertically orientated.

Comments: *Maofelis* is most similar to the primitive nimravid represented by a skull from Quercy (MA-PHQ 348) which has been identified originally as cf. *Eofelis* sp.^28^. This identification is not supported by our phylogenetic analysis, which did not place MA-PHQ 348 with *Eofelis* (see section on the phylogenetic analysis). MA-PHQ 348 is identified here as Nimravidae indet. Its similarity with *Maofelis* is based on plesiomorphic characters and does not suggest a close relationships between these taxa.

### Description

The rostral part of the skull is about 65% of the condylobasal skull length (Fig. 2; Supplementary Fig. 1). The braincase is about 43% of the condylobasal skull length. The rest of the skull is occupied by the orbitotemporal region. In lateral view, the dorsal margin of the skull is convex, with its highest point at the postorbital constriction. The greatest width across the postcanine teeth is 1.25 times greater than the greatest width across the braincase (posterior to the external acoustic meatus).

The external nasal aperture is 1.4 times taller than wide. In dorsal view, the lateral sides of the rostrum are subparallel, convex at the canine alveolus and concave between the canine alveolus and the anterior end of the zygomatic arch. The premaxillae project anteriorly beyond the anterior end of the nasals. The anterior end of the nasals is situated at the level of the middle of canine alveolus. The nasals are almost horizontal in lateral view and convex. The infraorbital foramen is large and placed at the root of the zygomatic process of the maxilla above the mesial root of P3.

The bony surface of the palate is poorly preserved. The maxillary-premaxillary suture is only partially preserved. The incisive foramina are relatively short and round, confined to the level of the canine distal half. Anteriorly, there are distinct parallel grooves leading to these foramina. The palatine extends anteriorly to the level of P3 protoconal swelling.

The zygomatic arch is gently curving, tall and narrow. In lateral view the zygomatic arch is slightly arching dorsally and placed along the ventral margin of the skull.

The orbital fossa is about half the length of, and lower than, the temporal fossa. The orbit is mostly open posteriorly, delimited by short postorbital processes of the frontal and zygomatic arch. The jugal contacts the lacrimal. The lacrimal foramen is posteriorly facing and not visible in lateral view.

The postorbital constriction is located at the middle of the temporal fossa, well posterior to the frontal postorbital process. The width at the postorbital constriction is about 50% of the width at the frontal postorbital processes (reconstructed).

There are prominent sagittal and nuchal crests on the braincase. The sagittal crest is very high, with a slightly convex dorsal margin. There are weak temporal ridges between the postorbital processes and the sagittal crest. The temporal ridges converge to form the sagittal crest posterior to the postorbital constriction.

The basicranium region is poorly preserved (see Supplementary Fig. 2). There are no bullar elements attached to the skull. The petrosal is placed in a deep depression and its ventral surface is distinctly dorsal to the ventral surface of the adjacent basisphenoid and basioccipital. There is no ventral process of the promontorium. The paroccipital process is short and placed just posterior to the mastoid region. The posterior lacerate foramen is anteromedial to the paroccipital process and confluent with the petrobasilar canal. A somewhat smaller hypoglossal foramen is posteromedial to the latter foramen. The posterior opening of the alisphenoid canal and the foramen ovale open in a common groove bordered by a ventromedial rim, just medial to the glenoid fossa. There is no postglenoid pedicle of the squamosal. The postglenoid foramen cannot be identified. There is no median ridge on the ventral side of the basioccipital. The basisphenoid and basioccipital have no lateral buttressing, suggesting that the bullar elements were not strongly attached to the basicranium, if present.

The choanae are similar in width to the foramen magnum. The pterygoid processes are converge slightly posteriorly.

The nuchal crest overhangs the occiput and extends posteriorly beyond the occipital condyles (Fig. 3). The occiput faces posteroventrally. The foramen magnum is wider than high and extends dorsally slightly beyond the dorsal margin of the occipital condyles.

The incisors are in gently curving arch and closely appressed (Fig. 4). There is a rather complete left I3 and badly damaged left I2 and right I2-3. The I1, judging from its alveolus, was somewhat smaller than I2. The I3 is about twice as large as I2. The crown of I3 is conical. There is a sizable diastema between I3 and C1.

The canine (length 16.6 mm, width 12.7 mm, measured at the enamel-dentine junction) is dagger-like, ventrally projecting, and moderately constricted laterally (length/width ratio is 1.31). The canine cross-section is asymmetrical, with the mesial carina placed at the mesiolingual corner, an almost flat lingual margin between the mesial and distal carinae, and a strongly convex lateral margin. Although the canine surface is poorly preserved, the small serrations along the distal carina can be recognized (supplementary Fig. 3). The right canine alveolus length is 18.3 mm, width is 14.3 mm. The postcanine teeth diverge posteriorly.

The P1 was small and one-rooted (Fig. 4). Its alveolus length is 3.4 mm (left) and 4.6 mm (right), and its alveolus width is 4.2 mm (left) and 3.5 mm (right). The P2 was two-rooted, with alveolus length 9.3 mm (left) and 9.8 mm (right) and alveolus width 5.8 mm (left) and 5.3 mm (right). The P3 is three-rooted (length 16.2 mm, 16.5 mm; width 11.0 mm, 10.8 mm for the left and right tooth respectively). The crown of P3 is submolariform, with a large paracone occupying half of the labial side and a blade-like metacone. The paracone and metacone are separated by a shallow notch. There is a large protoconal swelling with a small protocone. There is a distinct mesial cingulum but no parastyle. The P4 is a typical carnassial tooth with a large paracone, blade-like metacone, narrow talon, and small protocone placed mesiolingual to the paracone (length 19.8 mm, 20.3 mm; width 15.3 mm, 14.4 mm; metacone length 8.3 mm, 9.0 mm; protocone length 4.3 mm, 5.0 mm for the left and right tooth respectively). The paracone and metacone are separated by a shallow carnassial notch. There is no parastyle on P4.

There is a single molar, M1, preserved on the right side (length 6.4 mm; width 15.9 mm; Fig. 4). It has a typical tooth shape of the ultimate eutherian molar^43^,^44^,^45^, with a reduced metacone and metastylar blade and labially directed parastylar lobe. The paracone is relatively large. The talon has a distinct basin and a small protocone. The lingual margin of the M1 talon is placed more labial compared with the P4 talon. The M1 is two-rooted, with the lingual root larger.

For the skull measurements see Supplementary Fig. 4 and Supplementary Table 1.

## Discussion

### Saber-tooth specialization of *Maofelis*

**Figure 1:**
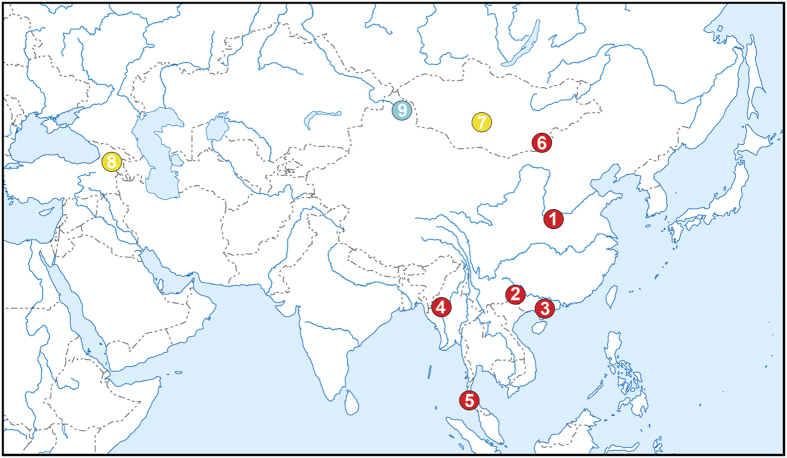
Map showing the known record of Nimravidae in Asia (red circles – Eocene, yellow – Oligocene, blue – Miocene). (1) Lushi Basin, Henan Province, China, Lushi Fm., middle Eocene, cf. *Eusmilus* sp., canine fragments^35^; (2) Bose Basin, Guangxi Province, China, Dongjin Fm., middle Eocene, *Hoplophoneus*? sp. or *Eusmilus*? sp., canine fragments^36^,^37^; (3) Maoming Basin, Guangdong Province, China, Youganwo Fm., middle-upper Eocene, *Maofelis cantonensis*, skull (this report); (4) Pondaung, Myanmar, Pondaung Fm., middle-upper Eocene, *Nimravus* sp., dentary fragment^34^; (5) Krabi Basin, Thailand, Formation B2, upper Eocene, *Nimravus* cf. *intermedius* and *Hoplophoneus* sp., maxilla and dentary fragments, isolated teeth^38^; (6) Khoer Dzan and Ergilin Dzo, Mongolia, Ergilin Dzo Fm., upper Eocene, *Nimravus intermedius* (=*N. mongoliensis*), dentary fragments^39^,^40^,^69^; (7) Tatal Gol and Taatsin Gol, Mongolia, Hsanda Gol Fm., lower Oligocene, *Nimravus mongoliensis* and Nimravidae indet., dentary fragments^16^,^48^,^68^; (8) Benara, Georgia, upper Oligocene, Nimravidae indet., isolated m1^41^; (9) Tieersihabahe, Xinjiang Uyghur Autonomous Region, China, Halamagai Fm., middle Miocene, Nimravidae indet., dentary fragment^42^. The map was generated by A. Averianov using Adobe Photoshop CS3 program.

**Figure 2:**
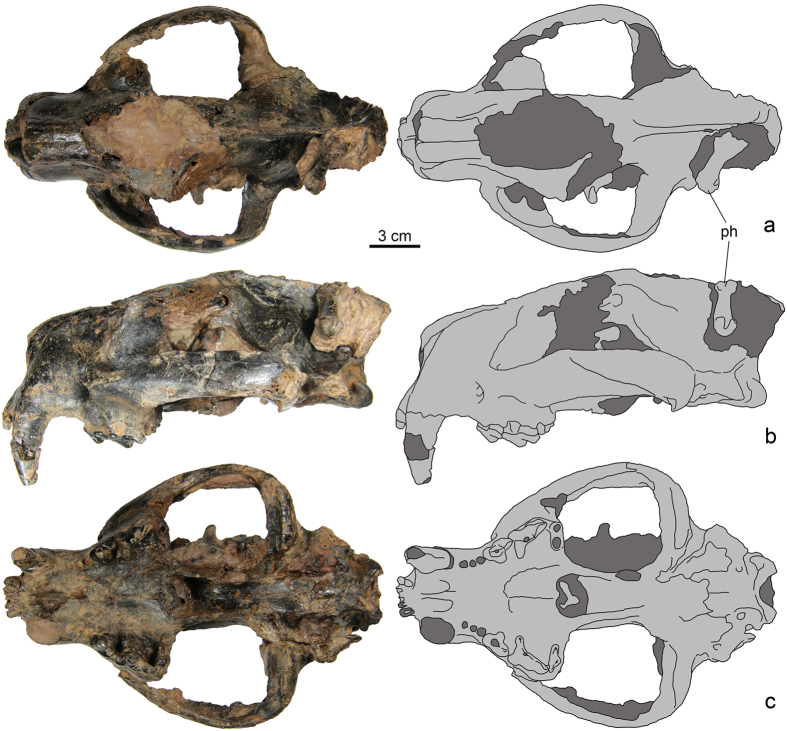
SYSU-M 2, holotype of *Maofelis cantonensis* gen. and sp. nov., in dorsal (a), lateral (b) and ventral (c) views, photographs and explanatory drawings. Abbreviation: ph, phalanx attached to the matrix.

**Figure 3:**
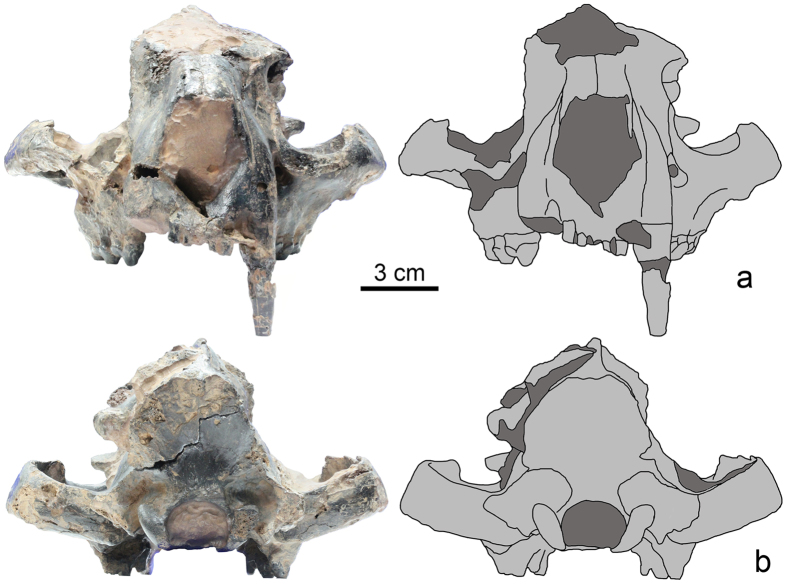
SYSU-M 2, holotype of *Maofelis cantonensis* gen. and sp. nov., in anterior (a) and posterior (b) views, photographs and explanatory drawings.

**Figure 4:**
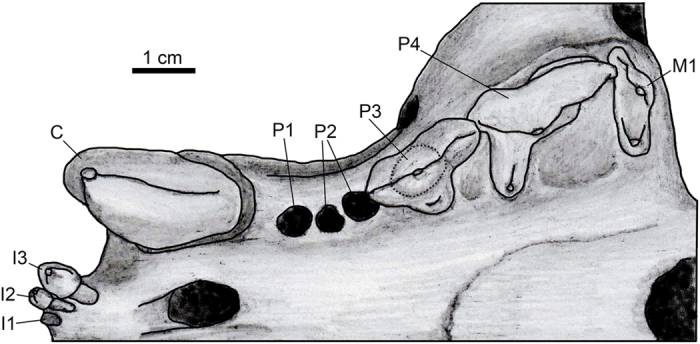
Reconstruction of upper dentition of SYSU-M 2, holotype of *Maofelis cantonensis* gen. and sp. nov., in ventral view. Drawing by A. Averianov.

The saber-tooth specialization of *Maofelis* is less pronounced compared with other known nimravid taxa. In *Maofelis* this specialization is expressed by a large and caniniform I3 and a relatively large upper canine which is only weakly laterally compressed (length/width ratio is 1.3) and has serrated distal carina. Most nimravid taxa have more compressed upper canines, with length/width ratio above 1.5. In most specialized taxa the upper canines are very flattened, with length/width ratio 2.7–3.2 2. The canine cross-section is similar to that of *Pseudaelurus* and intermediate between those of non-sabertooth and sabertooth felids^15^: Figs 1, 2, 3. The glenoid, mastoid, and paroccipital processes are all weakly developed in *Miaofelis*. The strong development of these processes is correlated with the ventral displacement of the mandibular articulation, which allows a wider gape needed for long upper canines, and development of powerful neck musculature^2^,^46^. The zygomatic arch of *Maofelis* is nearly horizontal in lateral view, while in derived nimravids it is strongly arched dorsally.

### Phylogenetic position of *Maofelis*

**Figure 5:**
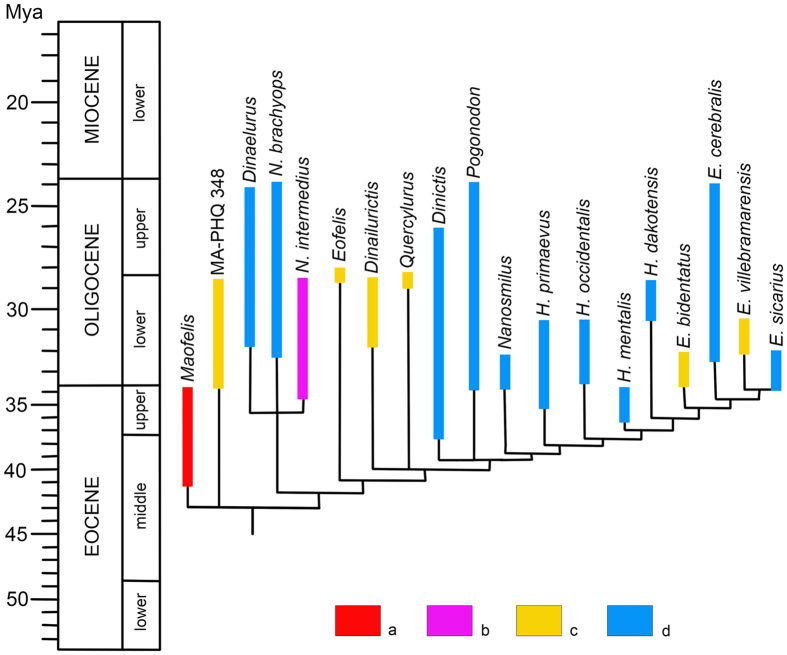
Phylogenetic tree of Nimravidae, modified by A. Averianov from Peigné^2^ according to the phylogenetic hypothesis presented herein. Distribution of taxa: (**a**) Asia; (**b**) Asia and Europe; (**c**) Europe; (**d**) North America. Abbreviations: E., *Eusmilus*; H., *Hoplophoneus*; N., *Nimravus*.

Our phylogeny of Nimravidae (Fig. 5 and Supplementary Fig. 5) is generally similar to that presented by Peigné^2^, differing in having greater resolution within the *Hoplophoneus*-*Eusmilus* clade, a more basal position for *Dinailurictis*, and absence of a sister group relationship between *Dinictis* and *Pogonodon*. *Hoplophoneus* is paraphyletic with regard to *Eusmilus*. *Maofelis* and Nimravidae indet. from Quercy (MA-PHQ 348) are the most basal nimravids, forming a polytomy with a clade containing all other nimravid taxa.

### Evolutionary history of Nimravidae

Nimravids represent the first radiation of cat-like hypercarnivores. They inhabited closed forest habitats and their decline in the late Oligocene in North America has been associated with the spread of grassland ecosystems^25^. The oldest known nimravids are Nimravidae indet. from the *Hoplophoneus*-*Eusmilus* clade from the middle Eocene Lushi and Dongjun formations in China^2^,^35^,^37^,^47^. The next oldest records are middle-late Eocene *Maofelis* reported here from the Youganwo Formation of South China (Supplementary Fig. 6) and *Nimravus* sp. from the Pondaung Formation of Myanmar^34^. *Nimravus* is also known from the late Eocene Ergilin Dzo Formation of Mongolia^39^,^40^,^69^. The middle-late Eocene Asiatic Nimravidae are already diversified, being represented by the less specialized *Maofelis*, moderately specialized *Nimravus*, and highly specialized members of the *Hoplophoneus*-*Eusmilus* clade. This suggests a long unrecognized history of Nimravidae in Asia and their possible origin on that continent. In North America, the oldest Nimravidae are the late Eocene *Dinictis* and *Hoplophoneus* (Fig. 5)^11^,^25^, which are relatively derived. In Europe Nimravidae, appeared only after the Grande Coupure event, in the early Oligocene (MP 21), and were represented by the most specialized known taxon, *Eusmilus*^2^,^28^. The next taxon to appear in Europe (early Oligocene, MP 22) is the relatively unspecialized *Nimravus*^2^. The early distribution of Nimravidae suggests that they migrated from Asia to North America in the late Eocene and to Europe in the early Oligocene. The second wave of nimravid migration from Asia to North America in the early Oligocene involved less specialized taxa from the *Dinaelurus*-*Nimravus* clade. In Europe during the early Oligocene there was a modest radiation of moderately specialized taxa (*Eofelis*, *Dinailurictis*, *Quercylurus*). The most specialized taxa from the *Hoplophoneus*-*Eusmilus* clade diversified mostly in North America (Fig. 5)^25^, with a dispersal to Europe (*E. villebramarensis*). Appearing and first diversifying in Asia during the middle-late Eocene, Nimravidae become very rare on that continent during the Oligocene. The Oligocene records of Asiatic nimravids come from the lower Oligocene Hsanda Gol of Mongolia^48^. *Nimravus* sp. from the late Oligocene Benara fauna in Georgia^41^ might be an European migrant. There is also a single nimravid record from the middle Miocene Halamagai Formation of North-West China^42^. The decline of Nimravidae in Asia during the Oligocene was likely caused by the earlier development of open habitats in Asia compared with North America^49^,^50^.

## Methods

### Geological settings

The Maoming Basin is an asymmetric northwest–southeast elongated upper Mesozoic–Cenozoic sedimentary basin nearly 40 km long and 16 km wide, located in southwest Guangdong Province, South China. The Upper Cretaceous–Neogene sedimentary sequence of the Maoming Basin is subdivided into Sanyajiang, Tongguling, Shangdong, Youganwo, Huangniuling, Shangcun, Laohuling, and Gaopengling formations^51^. All known vertebrate remains from the Maoming Basin were found in dark brown oil shales in the upper part of the Youganwo Formation, which were deposited in lacustrine conditions. The Youganwo Formation has been dated based on magnetostratigraphy and palynomorph assemblages as upper Eocene^52^,^53^, or middle-upper Eocene^54^. The vertebrate assemblage of the Youganwo Formation includes cyprinid fishes, carettochelyid, adocid, trionychid, and geoemydid turtles, tomistomine and alligatorid crocodiles, and chalicotherioid and amynodontid perissodactyls^55^,^56^,^57^,^58^,^59^,^60^,^61^,^62^,^63^,^64^,^65^.

### Phylogenetic analysis

For the phylogenetic analysis we used the data matrix presented by Peigné^2^ that included all nimravid genera. We changed the coding of character 24 (proportion and size of the protocone on M1) for MA-PHQ 348, a skull of primitive nimravid from Quercy, from “1” to “?” because M1 is absent on this specimen. *Maofelis* can be coded for 16 of the 33 characters (48.5%). The coding for *Maofelis* is the following: 0??0???001000???000101010????????. Ten thousands repetitions of the parsimony ratchet (island hopper) algorithm of NONA version 2.0 66 run with Winclada version 1.00.08 interface^67^ produced six most parsimonious trees with a length of 87 steps, a consistency index of 0.72, and a retention index of 0.89. The strict consensus of these six most parsimonious trees is present in Fig. 5 and supplementary Fig. 5.

## Additional Information

**How to cite this article**: Averianov, A. *et al.* First nimravid skull from Asia. *Sci. Rep.* **6**, 25812; doi: 10.1038/srep25812 (2016).

## Supplementary Material

Supplementary Information
