# Supplementary material for: First nimravid skull from Asia
Source: Sci Rep. 2016 May 10;6:25812. doi: 10.1038/srep25812 (PMC4861911; doi:10.1038/srep25812)
Supplement: Supplementary Information [file srep25812-s1.pdf]

Supplementary information to First nimravid skull from Asia by Alexander Averianov, Ekaterina Obraztsova, Igor Danilov, Pavel Skutschas and Jianhua Jin

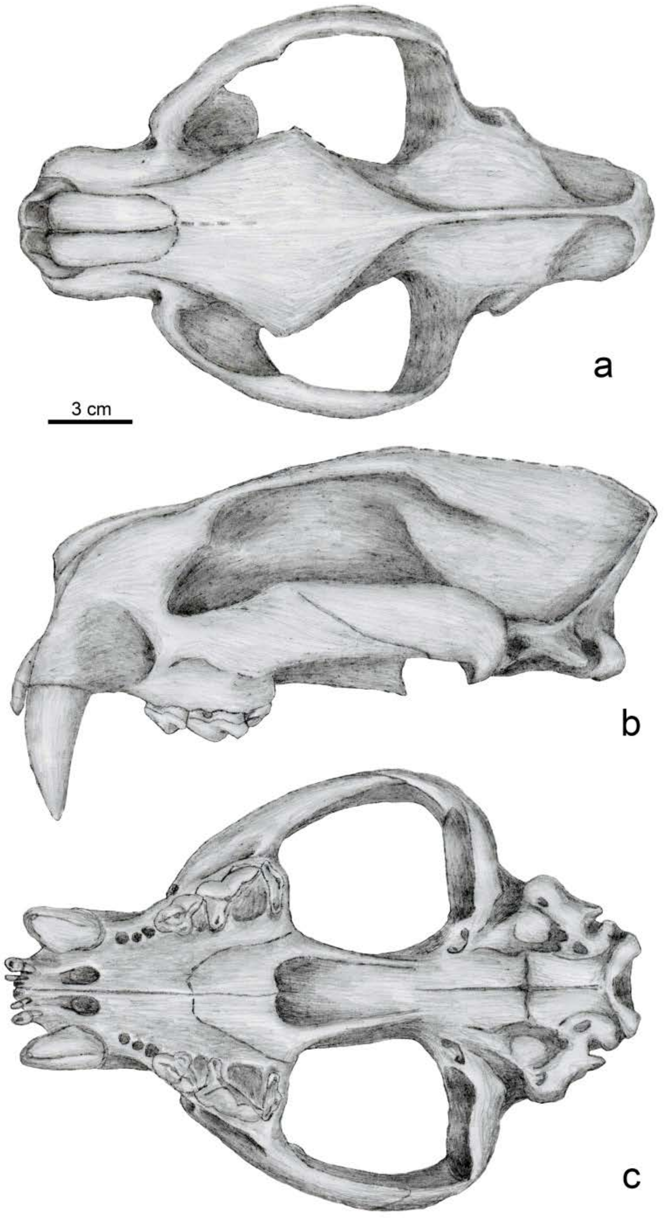

Supplementary Figure 1. Reconstruction of SYSU-M 2, holotype of *Maofelis cantonensis* gen. and sp. nov., in dorsal (a), lateral (b), and ventral (c) views. Drawings by A. Averianov.

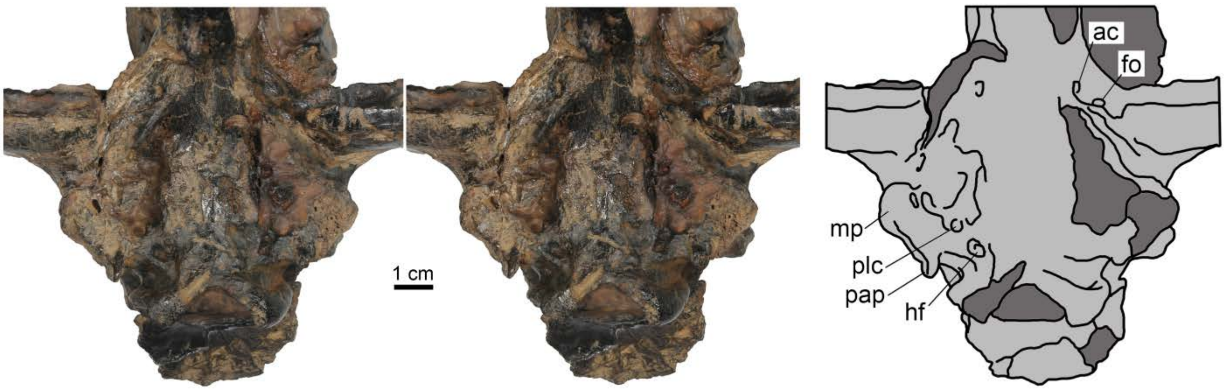

Supplementary Figure 2. SYSU-M 2, holotype of *Maofelis cantonensis* gen. and sp. nov., braincase in ventral view, stereopair and explanatory drawing. Abbreviations: ac, alisphenoid canal posterior opening; fo, foramen ovale; hf, hypoglossal fenestra; mp, mastoid process; pap, paroccipital process; plc, posterior lacerate foramen.

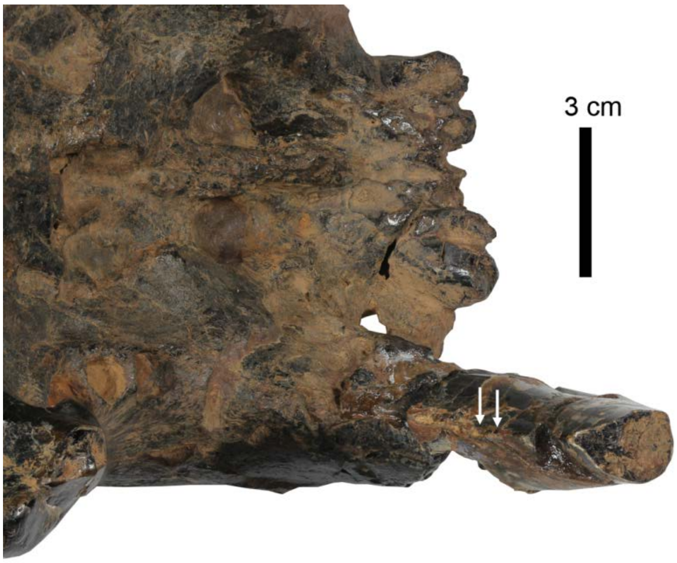

Supplementary Figure 3. SYSU-M 2, holotype of *Maofelis cantonensis* gen. and sp. nov., close up view of the canine distal carina with the preserved serrations indicated by arrows.

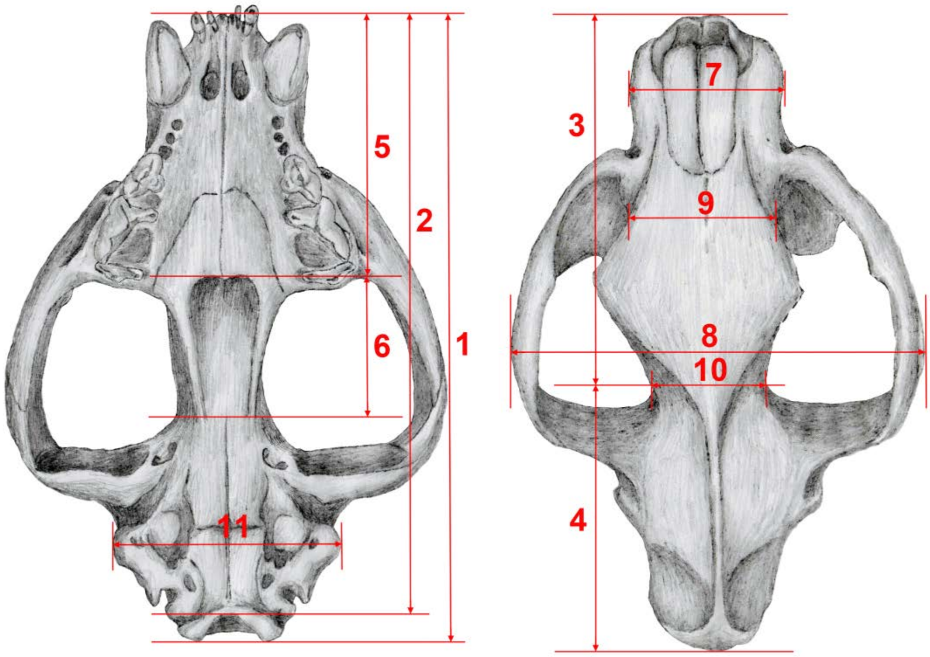

Supplementary Figure 4. Scheme of measurements of SYSU-M 2, holotype of *Maofelis cantonensis* gen. and sp. nov.

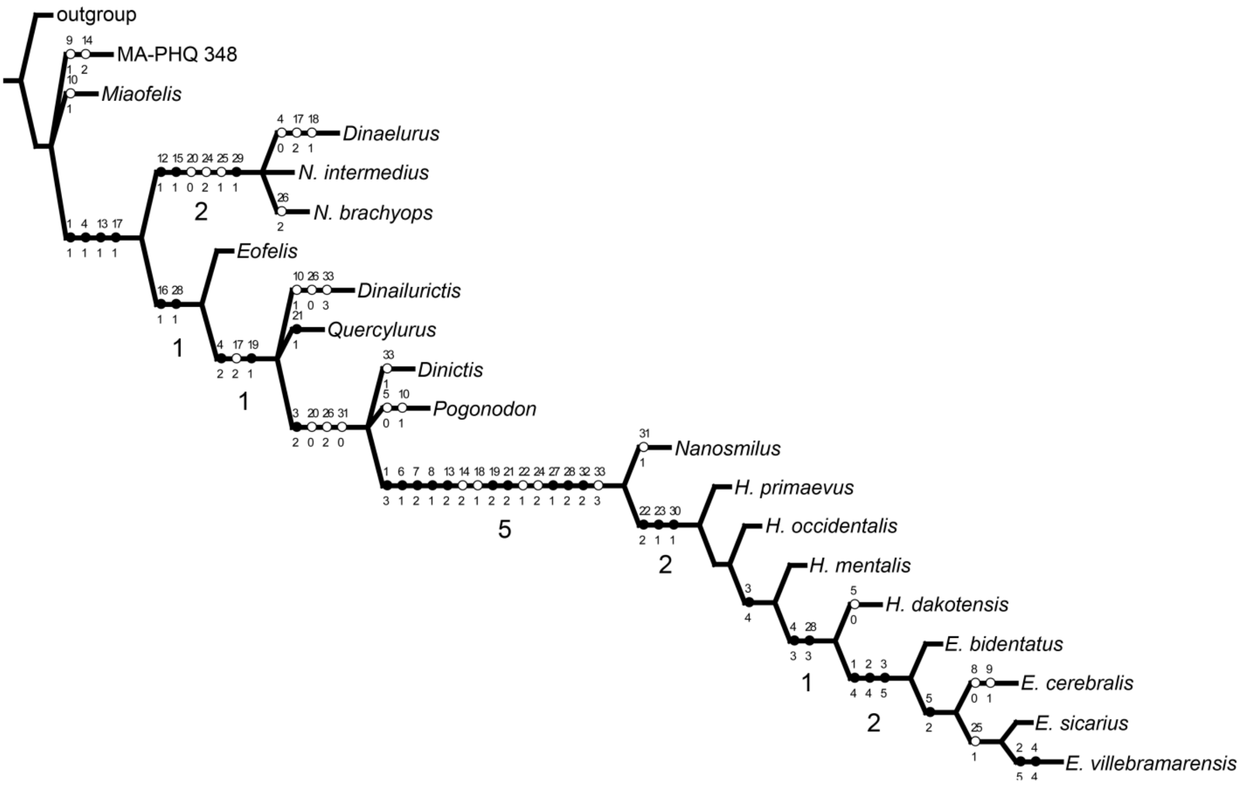

Supplementary Figure 5. Strict consensus tree of six most parsimonious trees produced by NONA ratchet algorithm using the data set presented by Peigné (2003) with addition of *Maofelis*. Only unambiguous characters are shown (white circles are homoplasies). The numbers at the circles are characters (above) and states (below). The large numbers below branches are Bremer support values found by TNT version 1.1 (Goloboff et al., 2003). Abbreviations: E. – *Eusmilus*; H. – *Hoplophoneus*; N. – *Nimravus*.

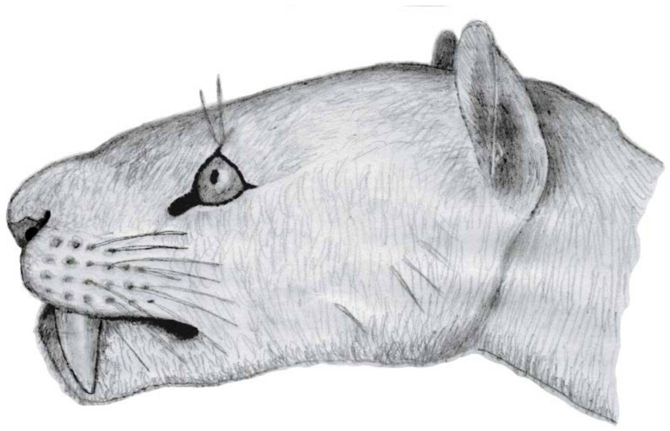

Supplementary Figure 6. Life reconstruction of *Maofelis cantonensis* gen. and sp. nov. Drawing by A. Averianov.

Supplementary Table 1. Skull measurements of SYSU-M 2, holotype of *Maofelis cantonensis* gen. and sp. nov. (in mm).

1. Condylobasal length	193.3
2. Basal length	183.4
3. Rostrum length	126.2
4. Braincase length	83.3
5. Bony palate length	83.1
6. Basipharyngeal canal length	125.0
7. Rostrum width at canines	50.0
8. Zygomatic width	139.9
9. Interorbital width	46.0
10. Width at postorbital constriction	33.9
11. Width at mastoid processes	69.8

References

Goloboff P.A., Farris J.S., Nixon K.C. 2003. Tree analysis using new technology. Program and documentation available from the authors (and at www.zmuc.dk/public/phylogeny).

Peigné S. 2003. Systematic review of European Nimravinae (Mammalia, Carnivora, Nimravidae) and the phylogenetic relationships of Palaeogene Nimravidae. Zoologica Scripta. **32**(3): 199-229.
